# Improving the strategy to identify historical military remains: a literature review and Y-STR meta-analysis

**DOI:** 10.1093/fsr/owad050

**Published:** 2023-12-25

**Authors:** Melinda R Mitchell, Janet Chaseling, Lee Jones, Toni White, Andrew Bernie, Larisa M Haupt, Lyn R Griffiths, Kirsty M Wright

**Affiliations:** Queensland University of Technology (QUT), Genomics Research Centre, Centre for Genomics and Personalised Health, School of Biomedical Sciences, Kelvin Grove, Queensland, Australia; Queensland University of Technology (QUT), Genomics Research Centre, Centre for Genomics and Personalised Health, School of Biomedical Sciences, Kelvin Grove, Queensland, Australia; Queensland University of Technology (QUT), Research Methods Group, Centre for Genomics and Personalised Health, School of Biomedical Sciences, Kelvin Grove, Queensland, Australia; Queensland University of Technology (QUT), Defence Innovation Hub, Centre for Genomics and Personalised Health, School of Biomedical Sciences, Kelvin Grove, Queensland, Australia; Unrecovered War Casualties-Army, Australian Defence Force, Russell Offices, Russell, Australian Capital Territory, Australia; Queensland University of Technology (QUT), Genomics Research Centre, Centre for Genomics and Personalised Health, School of Biomedical Sciences, Kelvin Grove, Queensland, Australia; Queensland University of Technology (QUT), Genomics Research Centre, Centre for Genomics and Personalised Health, School of Biomedical Sciences, Kelvin Grove, Queensland, Australia; Queensland University of Technology (QUT), Genomics Research Centre, Centre for Genomics and Personalised Health, School of Biomedical Sciences, Kelvin Grove, Queensland, Australia; Unrecovered War Casualties-Army, Australian Defence Force, Russell Offices, Russell, Australian Capital Territory, Australia; Royal Australian Air Force (RAAF), No 2 Expeditionary Health Squadron, RAAF Base Williamtown, Williamtown, New South Wales, Australia

**Keywords:** Y-STR, mutation rates, meta-analysis, Yfiler™, PowerPlex® Y23, historical identifications

## Abstract

The identification of historical military remains by Unrecovered War Casualties—Army (UWC-A) currently relies on Y-chromosome Short Tandem Repeat (Y-STR) testing when maternal relatives are not available, or when a mitochondrial DNA match does not provide sufficient certainty of identification. However, common Y-STR profiles (using Yfiler™) between sets of remains or families often prevent identification. To resolve these cases, an investigation of additional Y-DNA markers is needed for their potential inclusion into the DNA identification strategy. The number of genetic transmissions between missing soldiers and their living relatives needs to be considered to avoid false exclusions between paternal relatives. Analysis of 236 World War I/II (WWI/II) era pairs of relatives identified up to seven genetic transmissions between WWII soldiers and their living relatives, and nine for WWI. Previous Y-STR meta-analyses were published approximately 10 years ago when rapidly mutating markers were relatively new. This paper reports a contemporary literature review and meta-analysis of 35 studies (which includes 23 studies not previously used in meta-analysis) and 23 commonly used Y-STR’s mutation rates to inform the inclusion of additional loci to UWC-A’s DNA identification strategy. Meta-analysis found mutation data for a given Y-STR locus could be pooled between studies and that the mutation rates were significantly different between some loci (at *P* < 0.05). Based on this meta-analysis, we have identified two additional markers from PowerPlex® Y23 for potential inclusion in UWC-A’s identification strategy. Further avenues for potential experimental exploration are discussed.

**Key points:**

- From 236 UWC-A pairs of relatives, we observed up to nine genetic transmissions between WWI soldiers and their living relatives, and seven for WWII.
- MedCalc® software for meta-analysis utilizing the Freeman–Tukey transformation was run, which analysed 35 published studies and 23 commonly used loci. Previous Y-STR mutation rate meta-analyses are now 10 years old; this paper includes 23 studies that were not included in previous meta-analyses.
- Through meta-analysis, we identify two markers from PowerPlex® Y23 for potential inclusion in UWC-A’s historical remains identification strategy (alongside Yfiler™). We discuss potential next steps for experimental exploration of additional Y-DNA markers.

## Introduction

### DNA identification of historical military remains

Current DNA profiling methods for historical remains identification cases include mitochondrial DNA analyses and commonly used Y-chromosome Short Tandem Repeat (Y-STR) typing [1–10]. Tasked with the recovery and identification of missing Australian soldiers, Unrecovered War Casualties—Army (UWC-A) employ such methods in combination with historical and genealogical data, and, where possible, other scientific methods [11]. DNA is solely relied upon when other identifiers are unsuitable (e.g. paucity of antemortem dental records for WWI soldiers). Genealogical research traces familial lineages from missing soldiers to living maternal and paternal biological relatives, who may volunteer DNA samples for comparison against soldiers’ remains. Prior to this study, it was previously unknown how many genetic transmissions separated missing Australian WWI or WWII soldiers from suitable paternal donors.

Where possible, both maternal and paternal relatives of the soldier are located, and both lineages are investigated. When maternal relatives cannot be located, do not consent to providing their DNA for testing, or when a common mtDNA haplotype exists between multiple families, Y-STR analysis is relied upon to enable identification. Currently, UWC-A use the commonly used 17-locus Y-STR multiplex Yfiler™ for WWI and WWII investigations.

One problem faced when identifying historical remains recovered from battlefields, where hundreds or thousands of soldiers are still missing, is the occurrence of common Y haplotypes, meaning the profile may be the same between unrelated individuals. Low Y-STR discrimination power may also be encountered when only a partial DNA profile is obtained due to the degraded nature of the remains. In samples from a US population (African Americans, Asians, Hispanics, and Western European Caucasians; *n* = 1 032), Coble et al. [12] reported 15 haplotypes shared between “unrelated” men using the Yfiler™ multiplex. This increased to 70 common haplotypes when the number of loci decreased to a 12-locus multiplex, with one haplotype shared by 19 unrelated people. The current UWC-A identification strategy utilizing commonly used Y-STRs does not provide the discrimination power needed for common haplotypes encountered in Australian WWI/II cases. A contemporary analysis of mutation rates is needed to properly evaluate the inclusion of additional Y-STR loci into UWC-A’s DNA identification strategy.

### Rapidly mutating Y-STR markers

To increase haplotype discrimination between “related” men, PowerPlex® Y23 was developed, which contains two “rapidly-mutating” (RM) markers and four highly discriminating markers, as well as the original 17 Yfiler™ loci [13]. An RM locus can mutate 10 times faster (×10^−2^) than a standard Y-STR locus (×10^−3^) [14], and the discrimination capacity increased from 95.5% (developmental validation study; US population [15]) using Yfiler™ to 96% (worldwide study; 129 populations [16]) using the PowerPlex® Y23 system. These new markers are intended to resolve common haplotypes and distinguish between close relatives. This would be advantageous in cases where two or more close paternal relatives (e.g. between brothers or a father and a son) are suspected of being the donor of a questioned stain in a criminal investigation.

Using a panel of 13 RM markers, Ballantyne et al. [17] saw increased resolution of paternal lineages compared to the Yfiler™ multiplex. In the study, 66% of males could be discriminated from one another by mutation events detected using the RM panel, but this was the case for only 15% of males when using the Yfiler™ multiplex. From a sample size of 604 males, a total of three haplotypes were shared between eight individuals detected with the RM marker panel, and 33 haplotypes shared between 85 males when typed with the Yfiler™ multiplex. This demonstrates the capability of RM markers to resolve the common haplotypes of closely related individuals.

In the study presented here, we conduct a meta-analysis of 23 commonly used Y-STR loci across 35 studies to ultimately determine which, if any, additional commonly used Y-STR markers are suitable for inclusion into UWC-A’s historical military remains identification strategy. To build on previous meta-analyses [18], we included a subset of those originally analysed, in addition to 23 studies not included in previous analyses.

## Methods

### Estimating the number of genetic transmissions between paternal relatives

Family trees generated by UWC-A’s genealogist (194 WWI and 42 WWII pairs of relatives) were analysed to estimate the number of genetic transmissions likely between Australian soldiers and their living paternal relatives. The number of genetic transmissions for each family tree were manually counted and documented for subsequent analysis. The 95% confidence interval (CI) was calculated using Epitools, which maps input mean data to an appropriate *t*-distribution [19]; pre-set values for confidence level (0.95) and decimal places (one decimal place) were used, with the number of standard deviations set to three. The results of genetic transmission counting were then graphed using Microsoft Excel.

### Meta-analysis and associated analyses

#### Analyses between studies

To assess the suitability of Y-STR markers for historical remains identification cases, a meta-analysis-determined mutation rate was deemed an appropriate metric, as meta-analysis can take input data from numerous studies with differing sample sizes and, in this instance, provide a weighted estimate of a rate of mutation.

For the present meta-analysis, data were collated from 35 published studies of vastly different sample sizes [20–54] reporting mutation rates for Y-STR loci in the Yfiler™ and PowerPlex® Y23 multiplexes. Additional loci from the Yfiler™ Plus multiplex not in these multiplexes were not included in this study. Numerous loci in Yfiler™ Plus are also present in previous commercially available panels (Yfiler™ and PowerPlex® Y23), with additional loci being either RM or highly discriminating loci similar to those in the PowerPlex® Y23 multiplex. The suitability of RM markers needed to be further evaluated for the purpose of multigenerational military identifications. The authors of this paper acknowledge there are alternative ways to represent mutation rates; as the body of literature investigating Y-STR mutation rates uses scientific notation (e.g. ×10^−2^), this paper presents mutation rates in the same format. The authors also acknowledge there are numerous ways to calculate mutation rates, two of which are the Frequentist approach and the Bayesian approach. The software utilized for our meta-analysis, MedCalc® (v.20.009) [55], inputs data in the form of “positive” cases (number of mutations) and total cases (total number of meioses); this removes any potential differences between mutation rate calculation methods by calculating mutation rates using the same method before running meta-analysis calculations.

Meta-analysis on this data was performed with MedCalc® software (v.20.009) [55], which uses a Freeman–Tukey transformation (arcsine square root transformation) to calculate weighted summary proportions under the random effects model; this transformation helps by correcting for low (≤0.05) or high (≥0.95) frequencies, and small sample sizes (*n* ≥ 10) [56]. The meta-analysis gave each study a weighting when calculating frequency, with studies that had larger sample sizes given more statistical weighting than those with smaller sample sizes. MedCalc® software utilizes mutational data in the form of incidences of mutation and total number of genetic transmissions per locus. From there, the rate of mutation per locus was calculated, along with a confidence interval, and an I^2^ value. In meta-analyses, the I^2^ statistic is an indicator of heterogeneity, and reflects the proportion of the variance due to differences in effect size across studies rather than sampling error [57].

#### Analyses between Y-STR loci

Following inter-study analyses, general linear models were run using SAS software (v9.4) [58] to examine the variation in mutation rates between loci, with outputs generated including least squares mean (for effects: Locus and Study) mutation rates. General linear models were performed as the model determines if the dependant variable (in this instance, mutation rate) is affected by independent variables (e.g. locus, chromosomal banding, or study) [59].

A subset of the total 35 studies originally examined was refined based on which studies examined at least 20 of the PowerPlex® Y23 loci. Loci were ranked in order of equivalent mean mutation rate and grouped based on non-significance. The Levine’s test showed unequal variances of mutation rates between studies (*P* < 0.05); to adjust for this, the data were transformed using:

log(frequency + 0.1).

The Bonferroni correction was applied to account for multiple comparisons.

Previous literature has identified repeat complexity as one contributing factor to mutation rate variations between loci [32]. For this reason, General Linear Models analysis was conducted with the data for effects = Repeat Complexity and Chromosomal Band. Repeat complexity was determined using repeat region data where loci with a single repeat segment were deemed “simple”, and those with two or more were deemed “complex”. Testing to determine if chromosomal banding was a contributing factor involved grouping loci by chromosomal bands utilizing data from Hanson and Ballantyne [60] and Keerl [61].

## Results

### Estimating the number of genetic transmissions between paternal relatives

**Figure 1:**
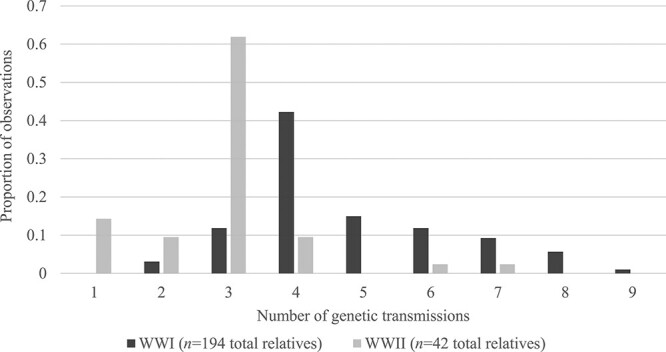
Number of genetic transmissions between WWI and WWII soldiers and a living paternal relative.

Figure 1 shows that most paternal relatives are four (95%CI: 3.6–4.4) genetic transmissions (42%) from a missing WWI soldier, and three (95%CI: 2.1–3.9) genetic transmissions (62%) from a missing WWII soldier. From the cases examined, up to nine genetic transmissions (2%) were identified between a WWI soldier and a paternal relative, and up to seven genetic transmissions (1%) were identified between a WWII soldier and a paternal relative.

### Meta-analysis and associated analyses

#### Analyses between studies

Data were summarized from published articles [20–54] for Yfiler™ and PowerPlex® Y23 loci; here, a total of 509 observations (estimates) were available for analysis. Owing to the fact many studies pooled the rates of the two DYS385 loci (DYS385a/DYS385b), these were also pooled in our analysis for consistency to previous literature. What was termed a “generation” by the authors of a particular study may have a longer time interval than others. As donors were mostly father–son pairs, a “generation” or “meiosis” can be termed one genetic transmission, and was considered as such for this analysis. Table 1 details meta-analysis data output including a point mutation rate estimate (with 95%CI) and a heterogeneity statistic.

**Table 1:** Detailed meta-analysis output of loci between studies.

**Locus**	**Number of studies**	**Number of genetic transmissions**	**Point estimate and 95%CI (random effects)**	**I** ^**2**^ **(heterogeneity, %)**	***P*-value^c^**	**Study references**
**DYS19** ^**a**^	31	25 784	2.32 × 10^−3^ (1.77 × 10^−3^–2.94 × 10^−3^)	0	0.514	[20–30, 32–35, 37, 38, 41–54]
**DYS385(a/b)** ^**a**^	28	38 368	3.41 × 10^−3^ (2.72 × 10^−3^–4.18 × 10^−3^)	20.15	0.172	[20–24, 26, 27, 30, 32–35, 37, 38, 41–54]
**DYS389I** ^**a**^	30	24 056	2.92 × 10^−3^ (2.28 × 10^−3^–3.64 × 10^−3^)	0	0.597	[20–28, 30, 32–35, 37, 38, 41–54]
**DYS389II** ^**a**^	30	24 061	4.14 × 10^−3^ (3.37 × 10^−3^–4.99 × 10^−3^)	0	0.615	[20–28, 30, 32–35, 37, 38, 41–54]
**DYS390** ^**a**^	31	25 331	2.91 × 10^−3^ (2.08 × 10^−3^–3.88 × 10^−3^)	31.43	0.050	[20–30, 32–35, 37, 38, 41–54]
**DYS391** ^**a**^	31	25 272	3.32 × 10^−3^ (2.47 × 10^−3^–4.29 × 10^−3^)	25.11	0.104	[20–30, 32–35, 37, 38, 41–54]
**DYS392** ^**a**^	31	25 202	8.90 × 10^−4^ (5.60 × 10^−4^–1.30 × 10^−3^)	0	0.468	[20–30, 32–35, 37, 38, 41–54]
**DYS393** ^**a**^	30	23 809	1.53 × 10^−3^ (1.08 × 10^−3^–2.87 × 10^−3^)	0	0.995	[20–28, 30, 32–35, 37, 38, 41–54]
**DYS438** ^**a**^	27	21 027	6.52 × 10^−4^ (3.52 × 10^−4^–1.04 × 10^−3^)	0	0.766	[20, 23–27, 30, 32–35, 37, 38, 41–54]
**DYS439** ^**a**^	27	20 991	5.43 × 10^−3^ (4.34 × 10^−3^–6.64 × 10^−3^)	14.60	0.250	[20, 23–27, 30, 32–35, 37, 38, 41–54]
**DYS437**	27	21 015	1.80 × 10^−3^ (1.27 × 10^−3^–2.42 × 10^−3^)	0	0.621	[20, 23–27, 30, 32–35, 37, 38, 41–54]
**DYS448**	20	17 455	1.14 × 10^−3^ (6.93 × 10^−4^–1.69 × 10^−3^)	0	0.536	[20, 27, 29, 30, 32, 34, 35, 37, 38, 41–51]
**DYS456**	20	17 480	5.27 × 10^−3^ (4.25 × 10^−3^–6.40 × 10^−3^)	0	0.731	[20, 27, 29, 30, 32, 34, 35, 37, 38, 41–51]
**DYS458**	20	17 499	8.38 × 10^−3^ (7.08 × 10^−3^–9.78 × 10^−3^)	0	0.955	[20, 27, 29, 30, 32, 34, 35, 37, 38, 41–51]
**DYS635**	21	18 225	3.82 × 10^−3^ (2.98 × 10^−3^–4.77 × 10^−3^)	0	0.621	[20, 27, 29, 30, 32, 34, 35, 37, 38, 41–51, 54]
**Y-GATA-H4**	23	18 618	2.14 × 10^−3^ (1.53 × 10^−3^–2.86 × 10^−3^)	0	0.558	[20, 23, 27, 29, 30, 32–35, 37, 38, 41–51]
**DYS481**	15	10 891	5.68 × 10^−3^ (4.36 × 10^−3^–7.18 × 10^−3^)	0	0.802	[31, 32, 36–38, 41–50]
**DYS533**	15	10 877	3.71 × 10^−3^ (2.65 × 10^−3^–4.93 × 10^−3^)	0	0.777	[31, 32, 36–38, 41–50]
**DYS549**	9	7 414	5.16 × 10^−3^ (3.60 × 10^−3^–7.00 × 10^−3^)	2.66	0.412	[31, 32, 36, 37, 41–43, 47, 49]
**DYS570** ^**b**^	17	12 392	9.95 × 10^−3^ (8.28 × 10^−3^–1.177 × 10^−2^)	0	0.520	[31, 32, 36–50]
**DYS576** ^**b**^	17	12 692	1.182 × 10^−2^ (1.002 × 10^−2^–1.377 × 10^−2^)	0	0.482	[31, 32, 36–50]
**DYS643**	9	7 503	1.13 × 10^−3^ (4.98 × 10^−4^–2.02 × 10^−3^)	0	0.999	[31, 32, 36, 37, 41–43, 47, 49]

^a^Original SWGDAM markers; ^b^Rapidly mutating markers; ^c^calculated from the Cochran’s Q (heterogeneity statistic) value given in the meta-analysis output. Cochran’s Q statistic is known for having problems with its statistical power, therefore it is often recommended to use an alpha value of 0.10 instead of the more commonly used 0.05.

For mutation rates from different studies for a given locus to be reliably pooled, meta-analysis should find low levels of heterogeneity (differences between the studies at a particular locus not due to random chance). A paper by Higgins et al. [62] suggested thresholds of “low” (25%), “medium” (50%), and “high” levels (75%) of heterogeneity to better interpret the validity of the pooled result. Table 1 summarizes the data of the current study, and shows I^2^ was relatively low, ranging between 0%–32%. From this, there is not sufficient evidence to suggest that a true difference exists between the studies for any locus, therefore mutation rates between studies for a given locus could be pooled. As meta-analysis did not find sufficient evidence to suggest published rates could not be pooled, a subset of studies was pooled together to determine if significant differences in mutation rate existed between other locus characteristics (e.g. repeat complexity and chromosomal banding).

#### Analyses between Y-STR loci

To best compare loci, the 13 studies that examined at least 20 of the PowerPlex® Y23 loci were utilized. Significant differences between groups of loci were determined from general linear models of equivalent mean mutation rates. Assessment of these general linear models (Tables 2–5) identified that:

(1) The equivalent mutation rates for RM markers (DYS570 and DYS576) were observed to be significantly different from over half of the loci in PowerPlex® Y23 (Table 2);
(2) The mutation rate for DYS533 and DYS643 (loci included in PowerPlex® Y23) were significantly lower than the RM markers;
(3) There were no significant differences in equivalent mutation rates between the 13 studies that examined 90% of PowerPlex® Y23 loci (Table 3);
(4) The equivalent mutation rates were significantly different between the Y-chromosomal band Yp11.2 and the other three bands (Table 4); and
(5) There was no equivalent mutation rate difference detected between simple and complex loci (Table 5).

**Table 2:** Ranked least squares mean mutation rates for 22 loci across 13 studies [32, 37, 38, 41–50] for: effect = locus; dependant variable = logfrequency.

**Locus**	**Equivalent mean mutation rate**	**Number of studies**	**Non-significant groupings (*P ≥* 0.05)**	
**DYS570** ^**b**^	1.058 × 10^−2^	13	*	
**DYS576** ^**b**^	9.602 × 10^−3^	13		
**DYS458**	6.274 × 10^−3^	13		#
**DYS549**	4.992 × 10^−3^	7		
**DYS456** ^**a**^	3.849 × 10^−3^	13		
**DYS389II** ^**a**^	3.790 × 10^−3^	13		
**DYS439** ^**a**^	3.355 × 10^−3^	13		
**DYS635**	2.872 × 10^−3^	13		
**DYS481**	2.754 × 10^−3^	13		
**DYS391** ^**a**^	2.268 × 10^−3^	13		
**DYS385(a/b)** ^**a**^	1.954 × 10^−3^	13	0.018	
**DYS389I** ^**a**^	1.857 × 10^−3^	13	0.011	
**DYS533**	1.580 × 10^−3^	13	0.002	
**DYS390** ^**a**^	1.402 × 10^−3^	13	0.001	
**Y-GATA-H4**	1.198 × 10^−3^	13	0.001	0.025
**DYS19** ^**d**^	1.084 × 10^−3^	13	0.001	0.010
**DYS437**	1.034 × 10^−3^	13	0.001	0.006
**DYS393** ^**a**^	9.66 × 10^−4^	13	0.001	0.003
**DYS392** ^**a**^	7.44 × 10^−4^	13	0.001	0.001
**DYS438** ^**a**^	5.89 × 10^−4^	13	0.001	0.001
**DYS448**	5.79 × 10^−4^	13	0.001	0.001
**DYS643**	5.49 × 10^−4^	7	0.001	0.001

Non-significant groupings at *P* ≥ 0.05 are non-significant when compared to that of marker indicated by either “*” or “#”. ^a^Original SWGDAM markers; ^b^Rapidly mutating markers.

**Table 3:** *P* values for multiple comparisons for: effect = study; dependant variable = logfrequency.

	[43]	[49]	[32]	[44]	[41]	[47]	[48]	[42]	[45]	[37]	[38]	[46]	[50]
[43]		1.000	0.842	0.172	0.086	1.000	1.000	1.000	1.000	0.575	1.000	1.000	1.000
[49]			1.000	1.000	1.000	1.000	1.000	1.000	1.000	1.000	1.000	1.000	1.000
[32]				1.000	1.000	1.000	1.000	1.000	1.000	1.000	1.000	1.000	1.000
[44]					1.000	1.000	1.000	1.000	1.000	1.000	1.000	1.000	1.000
[41]						1.000	1.000	1.000	1.000	1.000	1.000	1.000	1.000
[47]							1.000	1.000	1.000	1.000	1.000	1.000	1.000
[48]								1.000	1.000	1.000	1.000	1.000	1.000
[42]									1.000	1.000	1.000	1.000	1.000
[45]										1.000	1.000	1.000	1.000
[37]											1.000	1.000	1.000
[38]												1.000	1.000
[46]													1.000
[50]													

**Table 4:** Ranked least squares mean mutation rates for four chromosomal bands across 13 studies [32, 37, 38, 41–50].

**Locus**	**Chromosomal band**	**Equivalent mean mutation rate**	**Sample size** ^**c**^	**Yp11.2**	**Yq11.222**	**Yq11.221**	**Yq11.223**
**DYS19** ^**a**^ **DYS393**^**a**^ **DYS456**^**a**^ **DYS458** **DYS481** **DYS570**^**b**^ **DYS576**^**b**^	**Yp11.2**	4.146 × 10^−3^	91		*P* ≤ 0.001	*P* = 0.004	*P* ≤ 0.001
**DYS385a/b** ^**a**^ **Y-GATA-H4**	**Yq11.222**	1.501 × 10^−3^	65			*P* = 0.839	*P* = 1.000
**DYS389I** ^**a**^ **DYS389II**^**a**^ **DYS390**^**a**^ **DYS391**^**a**^ **DYS437** **DYS438**^**a**^ **DYS439**^**a**^ **DYS635** **DYS533** **DYS643**	**Yq11.221**	2.052 × 10^−3^	85				*P* = 0.064
**DYS392** ^**a**^ **DYS448** **DYS549**	**Yq11.223**	1.046 × 10^−3^	33				

^a^Original SWGDAM markers. ^b^Rapidly mutating markers. ^c^Sample size is the sum of the number of loci from all included studies in each band.

**Table 5:** Repeat complexity analysis across 13 studies [32, 37, 38, 41–50].

**Groups**	***P*-value**
Simple DYS19^a^ DYS389I^a^ DYS390^a^ DYS391^a^ DYS392^a^ DYS393^a^ DYS438^a^ DYS439^a^ DYS437 DYS456^a^ DYS458 Y-GATA-H4 DYS481 DYS533 DYS549 DYS570^b^ DYS576^b^ DYS643	0.142
Complex DYS385a/b^a^ DYS389II^a^ DYS448 DYS635	

^a^Original SWGDAM markers. ^b^Rapidly mutating markers.

To determine if chromosomal banding had any bearing on mutation rate differences, we repeated our analysis following locus grouping (Table 4). The mutation rates of loci in band Yp11.2 were significantly different (at *P* < 0.05) from Yq11.221, Yq11.222, and Yq11.223. Loci were then regrouped based on repeat complexity (Table 5), which was found to be non-significant (*P =* 0.142).

## Discussion

### Estimating the number of genetic transmissions between paternal relatives

As the main application of the newer “highly-discriminating” Y-STR multiplexes is for criminal casework (to make the distinction between close relatives who may be included as suspects), these methods only need to have markers that do not mutate between one to two genetic transmissions. Evidenced in Figure 1, the number of genetic transmissions possible in historical casework is (on average) three (2.1–3.9) to four (3.6–4.4), with up to nine possible genetic transmissions. This combined with the 10-fold increase in mutation rate of the RM loci demonstrates that they are unsuitable for use in historical military identifications. However, additional and accurate information on mutation rates and differences in other loci is imperative to inform additional locus choice to correctly resolve historical cases.

### Meta-analysis and associated analyses

As indicated in Table 1, low heterogeneity in mutation rates between studies for a given locus was observed. It is possible that complete resolution of heterogeneity may be accomplished through further information about the individuals used such as age, as well as additional mutation data from future research. However, there are conflicting opinions regarding whether the father’s age at their son’s birth is correlated with an increased rate of Y-STR mutation [20, 22, 25, 27, 32, 37]. As not all studies have disclosed the data, it is difficult to perform analysis with the inclusion of these characteristics. Nevertheless, the results presented thus far suggest that mutation rates for a given locus may be combined across studies, as meta-analysis revealed minimal variation between the studies for each locus. Furthermore, results from general linear models analysis did not suggest any significant differences in mutation rate existed between studies.

## Conclusion

This paper aimed to improve the DNA strategy for matching paternal relatives from unidentified historical military remains. This is the first study to investigate the number of genetic transmissions between missing Australian WWI and WWII soldiers with living paternal relatives (up to nine and seven genetic transmissions, respectively). Through this investigation, the need for further research into multigenerational Y-chromosomal family trees and the mutability of the markers used in historical identification cases has been highlighted. Through the evaluation of Y-STR markers (both standard and RM), this research has shed light on the significance this work could have on the outcomes of historical military identification casework both nationally and internationally. The correct identification of historical military remains is of the utmost importance, and by reviewing the literature and conducting our meta-analysis, we have brought the current bank of literature around this subject one step closer to the improvement of currently used methods.

A meta-analysis of commonly used Y-STR mutations rates (with these findings considered) confirms that RM markers are unsuitable for UWC-A cases, however, identified two additional markers (DYS533 and DYS643) for potential inclusion in the UWC-A DNA strategy. As these markers were included in the PowerPlex® Y23 due to their increased discrimination power, they may provide the extra discrimination power sufficient to identify historical military remains. Differences between mutation rates when loci were grouped by chromosomal location were observed; however, no differences between locus groups were observed when grouped by repeat complexity. The latter may be due to insufficient data, or no true difference existing between groups of loci when grouped by repeat complexity. As mutation rate differences were observed when loci were grouped based on chromosomal location, this should be taken into consideration when selecting additional Y-STR markers for inclusion in the historical military remains identification strategy.

Further work on Y-STR mutation rates in a (representative) WWI/II-era Australian sample is needed to determine the increase in discrimination power these markers may provide. Other avenues for experimental exploration could include the typing of uncommonly used Y-STR markers that may add discrimination power in this specific population, alongside other uncommonly used Y-DNA markers (such as Y-SNPs) with slower mutation rates [32, 63].
